# Minocycline Alleviates Cluster Formation of Activated Microglia and Age-dependent Dopaminergic Cell Death in the Substantia Nigra of Zitter Mutant Rat

**DOI:** 10.1267/ahc.20-00022

**Published:** 2020-11-21

**Authors:** Daisuke Taguchi, Ayuka Ehara, Taro Kadowaki, Shin-ichi Sakakibara, Kazuhiko Nakadate, Koichi Hirata, Shuichi Ueda

**Affiliations:** 1 Department of Judo Therapy, Faculty of Medical Technology, Teikyo University, Utsunomiya, Tochigi 320–8551, Japan; 2 Department of Histology and Neurobiology, Dokkyo Medical University School of Medicine, Mibu, Tochigi 321–0293, Japan; 3 Department of Neurology, Dokkyo Medical University School of Medicine, Mibu, Tochigi 321–0293, Japan; 4 Laboratory of Molecular Neurobiology, Institute of Applied Brain Sciences, Faculty of Human Sciences, Waseda University, 2–579–15 Mikajima Tokorozawa, Saitama 359–1192, Japan; 5 Department of Basic Science, Educational and Research Center for Pharmacy, Meiji Pharmaceutical University, 2–522–1 Noshio, Kiyose, Tokyo 204–8588, Japan

**Keywords:** dopamine neuron, activated microglia, minocycline, substantia nigra, pro-inflammatory cytokines

## Abstract

Microglial activation is a component of neurodegenerative pathology. Here, we examine whether activated microglia participate in age-related dopaminergic (DA) cell death in the substantia nigra pars compacta (SNc) of the zitter (*zi/zi*) rat, a mutant characterized by deletion of the attractin gene. Confocal microscopy with double-immunohistochemical staining revealed activated microglia-formed cell-clusters surrounding DA neurons in the SNc from 2 weeks after birth. An immunoelectron microscopic study showed that the cytoplasm of activated microglia usually contains phagosome-like vacuoles and lamellar inclusions. Expression levels of the pro-inflammatory cytokines *interleukin-1β* (*IL-1β*), *tumor necrosis factor-α* (*TNF-α*) and *inducible nitric oxide synthase* (*iNOS*) were increased in the midbrain of 2-month-old *zi/zi* rats. Chronic treatment with the anti-inflammatory agent minocycline altered the morphology of the microglia, reduced cluster formation by the microglia, and attenuated DA cell death in the SNc, and reduced the expression of *IL-1β* in the midbrain. These results indicate that activated microglia, at least in part and especially at the initial phase, contribute to DA cell death in the SNc of the *zi/zi* rat.

## I Introduction

The zitter (*zi/zi*) rat is characterized by a loss-of-function mutation with an 8-bp deletion at the splice donor site of *Attractin* (*Atrn*) [21] and exhibits several neuropathological changes, such as postnatal-onset of impaired oligodendrocyte differentiation [33], juvenile-onset of spongiform degeneration [18], and age-dependent degeneration of dopaminergic (DA) neurons in the substantia nigra (SN) [26, 37, 38]. There is a marked decrease in *Atrn* mRNA in the brains of *zi/zi* rats. The rat *Atrn* gene encodes two isoforms (a secreted and a membrane form) as a result of alternative splicing [35]. Transgenic rescue experiments indicate that the membrane-type *Atrn*, but not the secreted-type, is responsible for the *zi/zi* mutant phenotype and neuropathology [21]. Furthermore, the production of reactive oxygen species (ROS) and the levels of oxidative stress in the *zi/zi* brain are reportedly elevated [8, 9, 11, 33, 39]. The DA neurons in the SN of normal rats are characterized by high levels of *Atrn* expression [22, 27, 41]. In addition, an *in vitro* study by Paz *et al.* [29] demonstrated that knocking down *Atrn* expression exacerbates the degeneration of DA clone cells induced by 1-methyl-4-phenylpyridinium and lactacystin, whereas *Atrn* overexpression protects against the same insult. However, knocking down, *per se*, showed no immediate effect. Thus, it has been suggested that *Atrn* may play a neuroprotective role within DA neurons and that the lack of *Atrn* in DA neurons sensitizes them to neurotoxic substances.

Microglia are resident immune-competent cells in the brain. Under physiological conditions, microglia perform an immune surveillance role and sense the extracellular microenvironment [20]. The activation of microglia following infection or damage, or as observed in several diseases, leads to microglial proliferation and causes the microglia to become hypertrophic and produce a wide variety of pro-inflammatory and cytotoxic factors, including interleukin (IL)-1β, tumor necrosis factor (TNF)-α, and ROS [13, 36]. Interestingly, activated microglia are characterized by swollen cell bodies and shorter, thicker processes, and have been identified in the brains of *zi/zi* rats ranging in age from 2 weeks to 12 months [16]. In addition to DA neurons, an immunohistochemical study from this laboratory showed that 98% of microglia in normal Sprague-Dawley (SD) rats express Atrn [27]. Taken together, these observations suggest that a lack of Atrn in microglia triggers prolonged microglial activation generation of IL-1β and ROS, and leads to DA cell death in *zi/zi* rats.

Minocycline is a second-generation tetracycline that easily penetrates the blood-brain barrier and has powerful anti-inflammatory effects. Many *in vivo* studies have shown that minocycline treatment attenuates the activation of microglia and prevents the production of microglia-derived pro-inflammatory factors such as IL-1β and ROS [6, 15, 40].

In the present study, to elucidate whether activated microglia trigger DA cell death in the SN of zitter mutant rats, we examined the effects of chronic minocycline treatment on zitter rat microglia and DA neurons.

## II Materials and Methods

### Animals and treatment

One- and 2-week-old (W), and 2- and 6-month-old (M) homozygote zitter (*zi/zi*) and age-matched *zi/+* and Sprague-Dawley (SD) rats were used. Previous studies [33, 37, 38] and a preliminary study using the same microglial marker showed no significant neurochemical or morphological differences between heterozygote F1 progeny (*zi/+*) and normal SD rats. At postnatal day 10 (P10), *zi/zi* rats were divided into two groups and intraperitoneally treated with saline or minocycline-HCl (45 mg/kg; Sigma-Aldrich, St. Louis, MO, USA; minocycline-HCl was also gifted by Wyeth K.K. (Saitama, Japan; currently, Pfizer Inc.)), once a day for 20 consecutive days [40]. The remaining rats were further administrated minocycline-HCl (120 mg/kg/day in 5% sucrose water [6]) or 5% sucrose water by oral gavage until P60 (2M). To prevent side effects [12], the dose of minocycline in the present study was gradually reduced to 60 mg/kg/day (from P61 through P90; 3M) and 30 mg/kg/day (from P91 through P180; 6M). All rats used in the present study were raised and maintained in the Laboratory Animal Research Center, Dokkyo Medical University School of Medicine. All animals were housed in groups of two or three with continuous access to food and water, and were maintained on a 12 hr light/dark cycle. All procedures were approved by the Animal Welfare Committee of Dokkyo University School of Medicine and were in accordance with NIH guidelines.

### Single and double immunohistochemical staining

The animals were deeply anesthetized with sodium pentobarbital (50 mg/kg), then transcardially perfused with physiological saline followed by ice-cold fixative containing 4% paraformaldehyde and 0.2% picric acid in 0.1 M phosphate buffer (pH 7.4). Sectioning and immunohistochemical staining were conducted according to our previous studies [27, 37]. Briefly, the brains were cryoprotected with 30% sucrose, frozen with powdered dry ice, and serially cut into 30 μm sections with a cryostat (Type 2800N, Reichert-Jung, Nußloch, Germany). The sections were then incubated with polyclonal antiserum against ionized calcium binding adaptor molecule 1 (Iba1; as a marker for microglia) (1:2,000; Wako Pure Chem. Ind. Ltd., Osaka, Japan) and another series of sections were incubated with monoclonal antiserum for tyrosine hydroxylase (TH; as a marker for DA neurons) (1:20,000; Incstar, Stillwater, MN, USA). The specificity of each antibody used has been previously described [16, 26, 37]. Single immunohistochemical staining was visualized using the diaminobenzidine (DAB) method.

For double immunofluorescence staining, sections after incubation with Ibal and TH were incubated with the mixed secondary antibodies Alexa Fluor 568 goat anti-rabbit IgG (1:1,000, Molecular Probes, Eugene, OR, USA) and Alexa Fluor 488 goat anti-mouse IgG (1:1,000, Molecular Probes). After the nuclei were stained with DAPI (Molecular Probes), the sections were mounted on glass slides and immunofluorescent images were obtained under an FV500 confocal laser scanning microscope (Olympus, Tokyo, Japan). Three-dimensional images were composed of Z-stacks of twenty-five to thirty 0.6 μm thick optical slices, which were taken through entire microglia including the cell body and processes.

### Immunoelectron microscopy

The immunoelectron microscopical procedures were based on our previous methods [26, 27]. Briefly, 2M *zi/zi* rats treated with minocycline or vehicle control were deeply anesthetized with sodium pentobarbital (50 mg/kg i.p.), transcardially perfused with physiological saline, and finally perfused with ice-cold fixative containing 4% paraformaldehyde and 0.1% glutaraldehyde in 0.1 M PB. The brains were removed from the skulls and then post-fixed with the same fixative for 24 hr at 4°C. Sections 50 μm thick were cut on a VT1000S microtome (Leica Microsystems, Bensheim, Germany) and used for Iba1-immunoelectron microscopic analysis using the HRP-DAB method. After treatment with OsO_4_, the sections were dehydrated with alcohol and embedded in Epon-812 resin. Ultrathin sections were prepared with an ultramicrotome and examined with an electron microscope (JEOL, Tokyo, Japan).

### RNA extraction and quantitative PCR assay

Two-month-old *zi/zi* rats treated with minocycline or vehicle control, and age-matched SD rats, were analyzed. Each animal was euthanized, and the midbrain were dissected immediately. Tissues were snap frozen and stored at −80°C until RNA extraction. Total RNA isolated using TRIzol extraction (Life Technologies, Carlsbad, CA, USA) was reverse transcribed into cDNA using a ReverTra Ace qPCR RT kit (TOYOBO, Osaka, Japan) after DNase I (Takara Bio, Shiga, Japan) treatment, according to the manufacturer’s directions. The primers for *IL-1β*, *TNF-α* and *cyclophilin A* (*cyp A*) were synthesized according to the literature [30]. The sequences of the primers were as follows: *IL-1β*, forward 5'-cacctctcaagcagagcacag-3' and reverse 5'-gggttccatggtgaagtcaac-3' (78 bp; NW_047658); *TNF-α*, forward 5'-aaatgggctccctctcatcagttc-3' and reverse 5'-tctgcttggtggtttgctacgac-3' (110 bp; D00475); *cyp A*, forward 5'-tatctgcactgccaagactgagtg-3' and reverse 5'-cttcttgctggtcttgccattcc-3' (127 bp; NM_017101); *iNOS*, forward 5'-gggagccagagcagtacaag-3' and reverse 5'-catggtgaacacgttcttgg-3' (95 bp; NM_012611). For quantitative PCR, SYBR Premix Tx Taq (Takara Bio) was used according to the manufacturer’s instructions. We used ABI PRISM 7000 (Applied Biosystems Japan, Tokyo, Japan) for signal detection and analysis. Each target sequence was amplified during 40 cycles of PCR (denaturation at 95°C for 5 sec and annealing/extension at 59°C for 31 sec). For relative quantification of gene expression, each mRNA expression level was normalized to *cyp A* mRNA expression and compared using the comparative threshold cycle (ΔΔCt) method. Relative qualification was calculated using the formula 2^-ΔΔCt^.

### Data analysis

The total number of Iba1- or TH-immunoreactive cells in the SN pars compacta (SNc) was estimated by a modified stereological counting system using Stereo Investigator (Micro Bright Field Japan, Tokyo, Japan) [16, 39]. Immunopositive cell bodies were counted in specific areas. Every sixth section (30 μm thick, 180 μm apart) of the midbrain, including the intact SN, was processed for each immunocytochemistry. All data were compared by analysis of variance (ANOVA) followed by Scheffe or Bonferroni-Dunn tests using 0.05 levels of significance.

## III Results

### Clusters of activated microglia in the zitter SN are associated with dopaminergic neurons

**Fig. 1.**
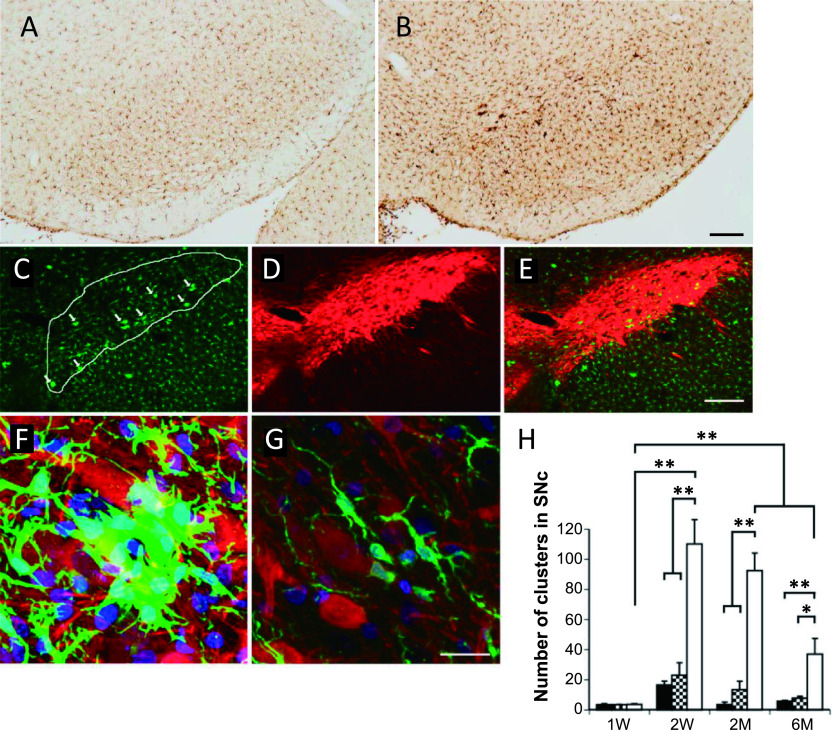
Immunohistochemical staining for Iba1 in the SNc at the age of 2M. (**A, B**) Distribution of Iba1 immunoreactive microglia in *zi/+* (**A**) and *zi/zi* (**B**) rats in low magnification photomicrographs. (**C–E**) Double immunohistochemical staining for Iba1 (green) and TH (red) in low magnification photomicrographs of the SNc in *zi/zi* rats. (**C**) Immunostaining for Iba1: the area of SNc is delineated by a white line. Clusters of Iba1-positive microglia evident in the SNc of the *zi/zi* rat are indicated with arrows. (**D**) Immunostaining for TH. (**E**) Merged image. (**F, G**) Three-dimensional confocal photomicrographs of the SNc of *zi/zi* rats. Clusters of Iba1-positive microglia surround the TH neurons in the SNc of *zi/zi* (**F**) but few are observed in *zi/+* (**G**) rats. The nuclei were stained using DAPI (blue). Bars = 200 μm (**A–E**) and 10 μm (**F, G**). (**H**) The number of Iba1-positive cell clusters in the SNc of SD (black bars), *zi/+* (checkerboard bars) and *zi/zi* (white bars) rats at different ages (n = 4 each group). * *P* < 0.05, ** *P* < 0.001.

**Fig. 2.**
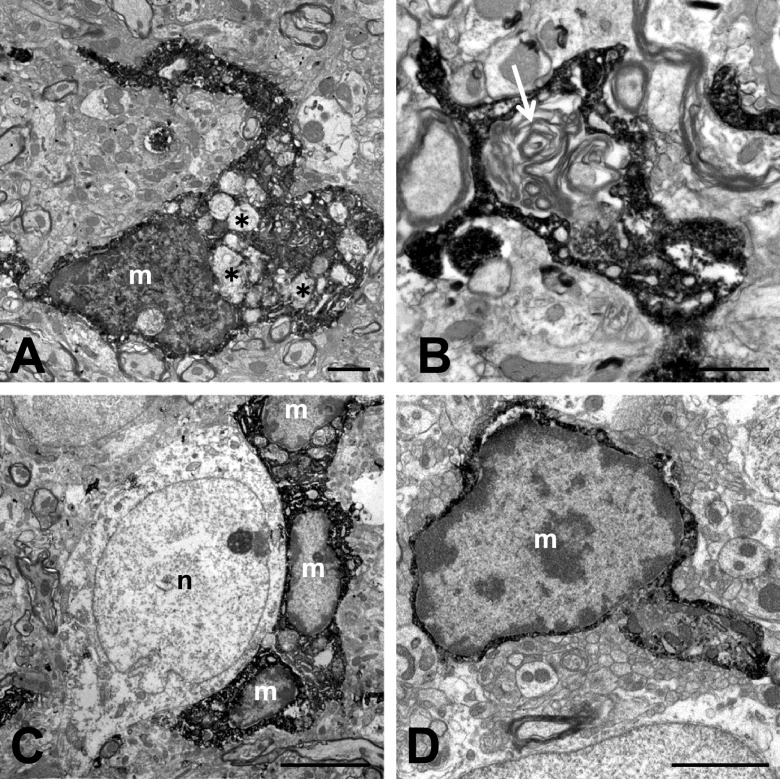
Ultrastructural characterization of Iba1-immunoreactive microglia (m) in the SNc of 2M *zi/zi* rats. (**A**) Microglia with thick enlarged processes in the neuropil of the SNc. The broadened cytoplasm of these cells contained several phagocytosis products (asterisks). (**B**) Activated microglia showing abnormal lamellar structures (indicated by an arrow). (**C**) Three microglia (m) with phagosome-like vacuoles of various sizes were observed adjacent to the neuron (n). (**D**) Iba1-immunoreactive microglia (m) in the SNc of *zi/zi* rats following minocycline administration for 2 months. Bars = 1 μm (**A, B**), 5 μm (**C**), and 2 μm (**D**).

Iba1-immunoreactive cell-clusters comprising at least five activated microglial cells [16] were more evident in the *zi/zi* rats than in the SD and *zi/+* rats, and were distributed throughout the midbrain, including the gray and white matter (Fig. 1A, B). The SNc of *zi/zi* rats more than 2 weeks old was unique with respect to the abundance of Iba1-immunoreactive cells (Fig. 1B, C, E). We conducted double-immunofluorescence staining with Iba1 and TH antibodies to identify whether the clusters of microglia in the *zi/zi* SNc were directly associated with the DA neurons. Confocal microscopic observations showed close association between the TH-immunoreactive neuronal elements (perikarya and/or dendrites) and the clusters of Iba1-immunoreactive cells in the SNc of *zi/zi* rats (Fig. 1F). In contrast, Iba1-immunoreactive cells with ramified morphology were sometimes associated with TH-immunoreactive perikarya in the SNc of *zi/+* rats (Fig. 1G). Two-factor factorial ANOVA for mean cell number of the clusters showed significant intergroup differences for age (F = 20.98, *P* < 0.0001) and group (F = 60.11, *P* < 0.0001), and a significant interaction between these two factors (age and group; F = 10.78, *P* < 0.001). *Post hoc* analysis indicated that the 2W, 2M and 6M *zi/zi* rats had significantly higher numbers of Iba1-immunoreactive clusters in the SNc than the SD and *zi/+* rats (Fig. 1H). To elucidate the fine structural features of the microglia from *zi/zi* rats, we examined the *zi/zi* SNc using immunoelectron microscopy. Iba1-immunoreactive cell bodies and processes were identified as having electron dense immunoreactive products within their cytoplasm (Fig. 2A, B). Iba1-immunoreactive cells were observed adjacent to neurons (perineuronal), as well as dispersed among the neuropils. The Iba1-immunoreactive cells had nuclei with electron-dense heterochromatin adjacent to the nuclear envelopes. The cytoplasm was filled with dense immunoreactive products, in addition to Iba1-negative cytoplasmic organelles such as the endoplasmic reticulum, Golgi complex and lysosomes. Interestingly, phagosome-like vacuoles of various sizes and with abnormal mitochondria and lamellar inclusions were observed within the cytoplasm of the cell body and processes (Fig. 2A–C).

### Minocycline attenuates microglial activation and dopaminergic cell death in the SN

**Fig. 3.**
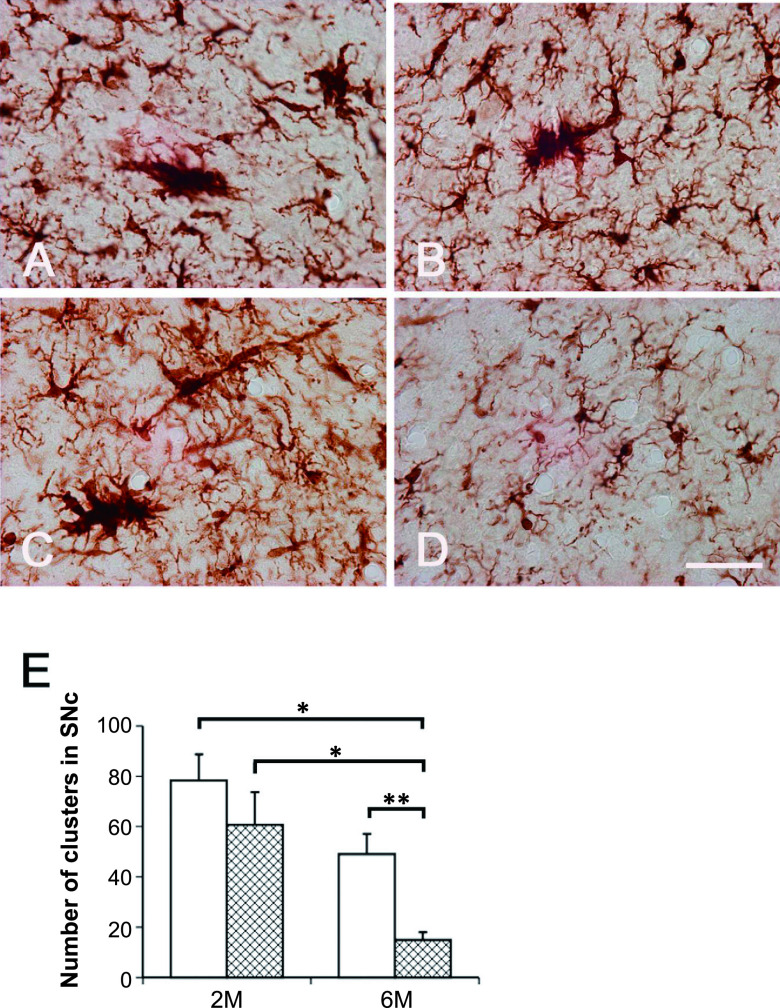
Effect of minocycline on the microglia in the SNc of *zi/zi* rats. (**A–D**) Iba1-immunoreactive microglia in vehicle- (**A, C**) and minocycline-treated (**B, D**) *zi/zi* rats for 2 months (**A, B**) and 6 months (**C, D**). Bar = 500 μm. (**E**) The number of Iba1-immunoreactive cell clusters in this area after different minocycline administration periods. The white bars show vehicle-treated *zi/zi* rats and the grid bars show minocycline-treated *zi/zi* rats (n = 3 each group). * *P* < 0.05, ** *P* < 0.01.

To determine whether minocycline prevents the observed increase in microglial clustering and morphological changes in the cells of *zi/zi* rats, we administered minocycline to *zi/zi* rats for 2 or 6 months and performed Iba1 immunohistochemistry analysis (Fig. 3A–D). After administration for 2 months, no morphological changes in Iba1-immunoreactive microglia were observed (Fig. 3A, B). Microglial morphology remained in the active form, and clusters were observed similar to in the vehicle control *zi/zi* rats. After minocycline administration for 6 months, Iba1-immunoreactive microglia appeared scattered throughout the neuropil of SNc, and most microglia exhibited morphologies typical of quiescent microglia, with a thin cell body and few processes (Fig. 3D). Immunoelectron microscopic examination of these microglia showed no lamellar inclusions but abnormal mitochondria in the cytoplasm (Fig. 2D). The number of Iba1-immunoreactive cell clusters in this area was significantly reduced in the 6M *zi/zi* rats treated with minocycline (Fig. 3E). Chronic administration of minocycline significantly reduced the number of activated microglia and also suppressed the formation of clusters.

**Fig. 4.**
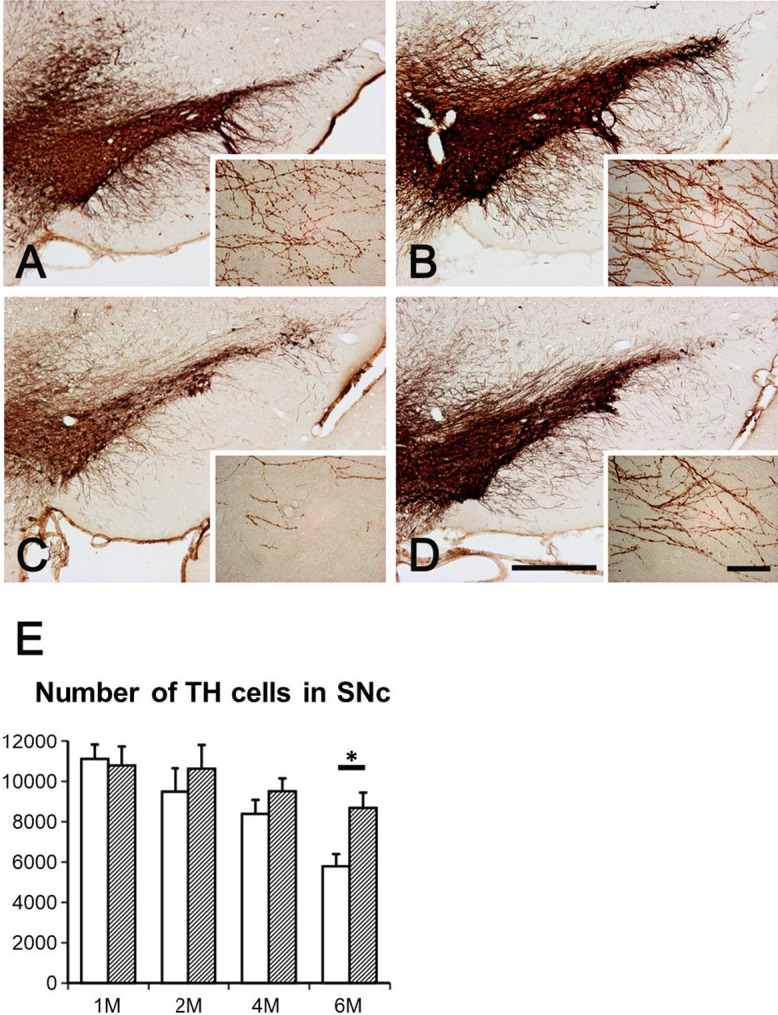
The effects of minocycline treatment on TH-immunoreactive neurons in the SN of *zi/zi* rats. (**A–D**) A comparison of TH-immunoreactive neurons in the SN of vehicle- (**A, C**) and minocycline-treated (**B, D**) *zi/zi* rats at 2M (**A, B**) and 6M (**C, D**). The right corners in panels **A–D** are high magnification images of the dendrites of TH-immunoreactive neurons in the SNr. Bars = 500 μm and 50 μm (corners). (**E**) The number of TH-immunoreactive neurons in the SNc from different age groups. The white bars show vehicle-treated *zi/zi* rats and the hatched bars show minocycline-treated *zi/zi* rats (n = 3 each group). * *P* < 0.005.

As shown in Figure 4, chronic administration of minocycline attenuated the loss of TH-immunoreactive neurons in the *zi/zi* SNc and TH-immunoreactive dendrites in the SN pars reticulata (SNr) (Fig. 4A–D). Two-factor factorial ANOVA analysis for TH-immunoreactive neurons in the *zi/zi* SNc showed significant differences between the different treatment groups (vehicle and minocycline; F = 11.29, *P* < 0.005) and also with respect to age (F = 11.68, *P* < 0.0001), but no significant interactions between these two factors was found (treatment and age; F = 0.96, *P* = 0.43) (Fig. 4E). *Post hoc* analysis revealed a significant difference in TH-immunoreactive neurons in the SNc of vehicle- and minocycline-treated 6M *zi/zi* rats. Taken together, these data suggest that minocycline attenuates microglial activation and dopaminergic cell death in the *zi/zi* SNc.

### Changes in the expression of *IL-1β*, *TNF-α* and *iNOS* in zitter rats after minocycline treatment

**Fig. 5.**
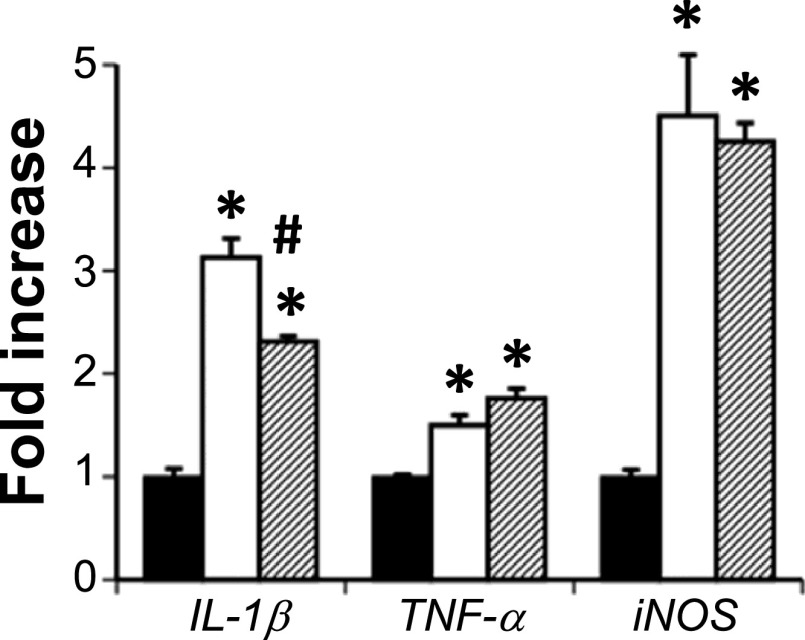
Expression of *IL-1β*, *TNF-α*, and *iNOS* mRNA in the midbrain of 2M SD (black bars), and *zi/zi* rats treated with vehicle (white bars) or minocycline (hatched bars) (n = 4 each group). The mRNA levels are presented as a fold change (mean ± SEM). * *P* < 0.01 vs. SD, # *P* < 0.01 vs. vehicle-treated *zi/zi* rats.

Our previous study [33] showed that the expression of *IL-1β* was significantly increased in *zi/zi* whole brain during early postnatal stages. As shown in Figure 5, *IL-1β*, *TNF-α* and *iNOS* mRNA were significantly upregulated in the midbrain of 2M *zi/zi* rats compared to the age-matched SD controls. The increase in *IL-1β* mRNA level was significantly inhibited by chronic minocycline treatment in the midbrain (62.5%), but there were no significant effects on *TNF-α* and *iNOS* mRNA expression following minocycline treatment (Fig. 5).

## IV Discussion

The present study indicates that activated microglia often cluster in the SNc of *zi/zi* rats. There is also elevated expression of pro-inflammatory cytokines and *iNOS* mRNA in the midbrain of *zi/zi* rats. In particular, microglial activation and clustering appeared to precede the reduction of DA neurons in the *zi/zi* SNc. Further, treatment with the anti-inflammatory drug minocycline significantly reduced the number of microglial cell clusters and alleviated age-related DA neuronal cell death in the *zi/zi* SNc.

The activated microglia frequently fused to form cell clusters in the *zi/zi* SNc and were observed as early as during the first 2W, and the number of these clusters peaked at 2W. Thereafter, the number of clusters appeared to gradually reduce but *zi/zi* greater than two weeks of age still had many clusters in comparison with age-matched *zi/+* and SD. The confocal and immunoelectron microscopy studies provided the characteristics of activated microglia in the *zi/zi* SNc. The activated microglia had swollen cytoplasms with phagocyte-like vacuoles of various sizes and with different inclusions. These microglia were assumed to be in stage three of microglial activation, the phagocytosis stage [32]. These activated microglia also expressed lysosome membrane antigen ED1, a specific marker for phagocytic microglia and macrophages (data not shown).

Microglial activation and clustering has been observed in the SN of animal models for Parkinson’s disease (PD) [4, 15, 23, 40]. In PD, activated microglia actively cluster around dystrophic DA neurons and contribute to DA cell death [36]. Our previous histochemical study failed to detect any cells labeled with Fluoro-Jade C, a sensitive and reliable marker for the specific localization of degenerative neuronal cells [34], in the *zi/zi* SNc until 4M [7]. A previous immunoelectron microscopic study showed that 4M *zi/zi* SNc neurons presented with several features of cellular injury, such as numerous lipofuscin granules, enlarged Golgi apparatus, and abnormal mitochondria that were still TH-immunoreactive [26]. Thus, some *zi/zi* TH-immunoreactive DA neurons exhibiting clusters of activated microglia may be in a state of ongoing degeneration. Taken together, the degeneration process of DA neurons in the *zi/zi* SNc is likely initiated in 2M *zi/zi* rats and slowly progresses with age. Activation of the microglia initiates the loss of TH-immunoreactivity in DA neurons of the *zi/zi* SNc.

The present data demonstrate that the expression of *IL-1β*, *TNF-α* and *iNOS* mRNAs increased in the midbrain of 2M *zi/zi* rats. At 6M, these increased expressions decreased to the same levels as those of control SD [14]. This study shows that chronic administration of minocycline reduces the expression of *IL-1β* mRNA in the midbrain at 2M and attenuates the loss of DA neurons in the *zi/zi* SNc. Systematic IL-1β administration exacerbates the degeneration of DA neurons and microglial activation in the SN [31], and administration of an IL-1 receptor antagonist attenuates the loss of DA neurons caused by lipopolysaccharide (LPS)-induced sensitization to the degeneration of DA neurons [19]. Taken together, it is likely that minocycline may, at least in part, attenuate this mutation-induced DA cell death in the SNc by inhibiting microglial activation and reducing *IL-1β* expression. However, minocycline had no effect on the expression of *TNF-α* and *iNOS* mRNAs. Biscaro *et al.* [1] reported that chronic minocycline treatment attenuated microglial activation histologically but TNF-α inhibition was not significant whereas IL-10, which is a downregulator of TNF-α, increased. These findings suggest that minocycline treatment may indirectly suppress TNF-α without significantly inhibiting expression. Similarly, our findings indicate that *TNF-α* and *iNOS* may be indirectly suppressed even if their expression is not significantly suppressed. Further studies are required to elucidate the regulatory mechanism by which expression is altered by chronic minocycline treatment.

In general, activated microglia show opposing properties: the phagocytic property has a neuroprotective role by destroying invading microorganisms and removing deleterious debris, whereas the cytotoxic property acts by releasing neurotoxic substances [20]. Recently, two microglial phenotypes have been proposed based on analogies of several markers. The M1 phenotype is cytotoxic and exhibits pro-inflammatory markers such as IL-1β, and the M2 phenotype contributes to repair and regeneration by expressing anti-inflammatory markers such as TGF-β and Cox2 [3, 10]. Based on these criteria, most microglia in the SNc of *zi/zi* rats are of the M1 phenotype.

Interestingly, reactive astrocytes also produce *IL-1β* [5]. Neuropathological changes to astrocytes have been noted in the *zi/zi* rat [17] and thus the elevated expression of *IL-1β* mRNAs could be attributed to the reactive astrocytes within the midbrain of this mutant. Elevated levels of ROS released from activated microglia, reactive astrocytes and SN neurons could synergistically damage the nigrostriatal DA pathway, leading to age-dependent neuronal loss in the SNc of *zi/zi* rats.

It was previously demonstrated that *zi/zi* rats, a loss-of-function mutant of *Atrn*, exhibit impaired oligodendrocyte differentiation in early development [33] and degeneration of the nigrostriatal DA system during aging [9, 26, 37] through microglial activation with high expression of cytokines and ROS. IL-1β, TNF-α and ROS are pleiotropic functional molecules responsible for a diverse range of signaling events within cells, leading to cell death [2, 24, 28]. Atrn plays a role in anti-oxidant signals in neurons via the extracellular signal-related kinase (ERK)-mediated cell survival pathway [8, 25, 29]. Although we could not identify the signal pathways for microglial activation, Atrn may be involved in the intracellular inactivation mechanisms of microglia. Pathogenesis in *zi/zi* rats may involve microglia-induced cytokines and ROS, in addition to oxidative stress arising from the metabolic- and auto-oxidation of DA neurons. The exact molecular mechanisms by which Atrn exerts anti-oxidant and/or anti-inflammatory effects remain to be elucidated.

## V Conflicts of Interest

The authors declare that they have no conflicts of interest.

## VI Acknowledgments

The authors would like to thank Ms. Shukuko Minami for technical assistance, Ms. Fusae Terauchi for assistance in preparing the manuscript, and FORTE Science Communications for English language editing.
